# Severe Alcohol-Associated Lactic Acidosis Presenting With Extreme Hyperlactataemia: A Case Report

**DOI:** 10.7759/cureus.113082

**Published:** 2026-07-21

**Authors:** Madhupawani Wijayasuriya Wijayasuriya Arachchige, Kyaw Thiri Tun, Chathura Madhushan Angulugaha Angulugaha Gamage, Taha Elsahy

**Affiliations:** 1 Acute Medicine, Peterborough City Hospital, Peterborough, GBR; 2 Geriatric Medicine, Peterborough City Hospital, Peterborough, GBR

**Keywords:** acute alcohol intoxication, alcohol-associated lactic acidosis, alcoholic ketoacidosis, alcohol use disorder, ethanol toxicity, high anion gap metabolic acidosis, ketoacidosis, severe hyperlactataemia

## Abstract

Alcohol-associated lactic acidosis (AALA) is a rare but potentially life-threatening complication of acute or chronic alcohol exposure. Severe hyperlactataemia attributed primarily to alcohol is uncommon and should prompt careful evaluation for alternative or co-existing causes, including sepsis, hypoperfusion, toxic ingestion, seizures and ischaemia.

We report the case of a 42-year-old man who presented to the emergency department with generalised malaise and vomiting following one week of heavy alcohol consumption involving whisky and beer. He had a history of recurrent hospital admissions related to alcohol intoxication but no significant documented medical comorbidities. Initial venous blood gas analysis revealed profound high anion gap metabolic acidosis, with a pH of 6.86, bicarbonate of 5.9 mmol/L, anion gap of 40 mmol/L, and lactate greater than 20 mmol/L. Serum ethanol concentration was elevated at 297 mg/dL, and blood ketones were markedly elevated at 6.5 mmol/L. Initial investigations showed mild leucocytosis, a normal C-reactive protein level, mildly elevated serum creatinine with preserved estimated glomerular filtration rate, and transaminitis with aspartate transaminase predominance. Paracetamol and salicylate levels were negative, methanol was within the laboratory reference range, and contrast-enhanced computed tomography of the chest, abdomen, and pelvis did not identify an infective, ischaemic, or intra-abdominal cause.

The patient was admitted to the intensive care unit and managed supportively with intravenous fluids, intravenous thiamine supplementation, and close biochemical monitoring. He showed rapid clinical and metabolic improvement, with lactate decreasing to 4.6 mmol/L by 10 hours and normalising by 16 hours. He remained stable without vasopressor support, ventilatory support, or renal replacement therapy and was discharged home on day four.

This case highlights the importance of considering AALA with concomitant ketoacidosis in patients with severe high anion gap metabolic acidosis and significant alcohol exposure. Early recognition, systematic evaluation for life-threatening alternative diagnoses, and supportive treatment with fluid resuscitation and thiamine replacement may be associated with rapid clinical and biochemical recovery.

## Introduction

Lactic acidosis is a common metabolic disturbance in acutely unwell patients and may occur in association with tissue hypoperfusion, sepsis, hypoxia, seizures, drugs or toxins, hepatic dysfunction, or impaired mitochondrial metabolism [1]. Alcohol-associated lactic acidosis (AALA) is a less frequently recognised cause of severe hyperlactataemia and is generally considered a form of type B lactic acidosis when the degree of lactate elevation occurs without overt systemic hypoperfusion sufficient to explain the metabolic disturbance [1,2].

Severe hyperlactataemia directly attributable to acute ethanol intoxication alone is uncommon. In patients with significant alcohol exposure, marked lactate elevation should therefore prompt careful assessment for alternative or co-existing causes [3]. This creates an important diagnostic challenge: alcohol exposure may contribute to lactic acidosis, but clinicians should avoid prematurely attributing profound high anion gap metabolic acidosis to alcohol without first evaluating for immediately life-threatening conditions, including sepsis, shock, seizures, ischaemia, renal or hepatic failure, salicylate toxicity, metformin-associated lactic acidosis, and toxic alcohol ingestion [1,4-6]. A lactate concentration greater than 5 mmol/L is commonly used to define severe lactic acidosis and carries prognostic significance in critically ill patients [1,2].

We present a case of profound high anion gap metabolic acidosis, with lactate greater than 20 mmol/L, in a patient with heavy alcohol intake, preserved blood pressure, normal oxygen saturation, negative toxicology screening for common co-ingestions, negative blood cultures, a normal contrast-enhanced computed tomography scan of the chest, abdomen, and pelvis, and rapid recovery following supportive treatment with intravenous fluids and thiamine replacement.

## Case presentation

A 42-year-old man was brought to the emergency department with generalised malaise and vomiting following one week of heavy alcohol consumption, reportedly involving multiple bottles of whisky and beer. He denied fever, seizures, abdominal pain, or medication overdose. His medical history was significant for recurrent hospital admissions related to alcohol intoxication. He had no other documented chronic medical comorbidities and was not known to be taking regular medication.

On arrival, his Glasgow Coma Scale score was 15/15. His heart rate was 114 beats/min, blood pressure was 136/84 mmHg, respiratory rate was 26 breaths/min, oxygen saturation was 98% on room air, and temperature was 36.8°C. Capillary refill time was normal, and peripheral pulses were well felt. Cardiovascular, respiratory, abdominal, and neurological examinations were otherwise unremarkable, with no abdominal tenderness, peritonism, focal neurological deficit, or clinical evidence of infection.

Initial venous blood gas analysis showed profound high anion gap metabolic acidosis, with a pH of 6.86, bicarbonate of 5.9 mmol/L, anion gap of 40 mmol/L, and lactate greater than 20 mmol/L. Serum ethanol concentration was markedly elevated at 297 mg/dL. Initial laboratory investigations showed mild leucocytosis and a normal C-reactive protein level. Serum creatinine was mildly elevated, with a preserved estimated glomerular filtration rate. Liver biochemistry showed transaminitis with aspartate transaminase predominance, while bilirubin, albumin, and coagulation profile were preserved. Blood glucose was normal, while blood ketones were markedly elevated. Paracetamol and salicylate levels were negative, and methanol was within the laboratory reference range. The initial laboratory findings are summarised in Table 1.

**Table 1:** Initial laboratory investigations on admission eGFR: estimated glomerular filtration rate; INR: international normalised ratio; APTT: activated partial thromboplastin time; HbA1c: glycated hemoglobin

Test	Result	Reference range
White blood cells	13.1 × 10⁹/L	4.0-11.0 × 10⁹/L
Haemoglobin	159 g/L	130-180 g/L
Platelets	206 × 10⁹/L	150-400 × 10⁹/L
C-reactive protein	1 mg/L	<5 mg/L
Serum creatinine	119 μmol/L	59-104 μmol/L
eGFR	65 mL/min/1.73 m²	>60 mL/min/1.73 m²
Urea	7.0 mmol/L	2.5-7.8 mmol/L
Serum sodium	138 mmol/L	133-146 mmol/L
Serum potassium	4.5 mmol/L	3.5-5.3 mmol/L
Serum magnesium	1.04 mmol/L	0.70-1.00 mmol/L
Serum calcium	2.26 mmol/L	2.20-2.60 mmol/L
Serum phosphate	0.84 mmol/L	0.80-1.50 mmol/L
Alanine transaminase	193 IU/L	<41 IU/L
Aspartate transaminase	514 IU/L	10-50 IU/L
Total bilirubin	11 μmol/L	0-21 μmol/L
Albumin	46 g/L	35-50 g/L
Uric acid	713 μmol/L	200-430 μmol/L
Amylase	109 IU/L	<100 IU/L
Creatine kinase	115 IU/L	40-320 IU/L
INR	0.86	0.80-1.25
APTT	22.9 s	24-36 s
Paracetamol	<5 mg/L	<5 mg/L
Salicylate	<4 mg/L	Not detected
Ethanol	297 mg/dL	<10 mg/dL
Methanol	<100 mg/L	<100 mg/L
Blood glucose	4.3 mmol/L	3.5-7.8 mmol/L
Blood ketones	6.5 mmol/L	<0.6 mmol/L
HbA1c	43 mmol/mol	20-42 mmol/mol

**Figure 1:**
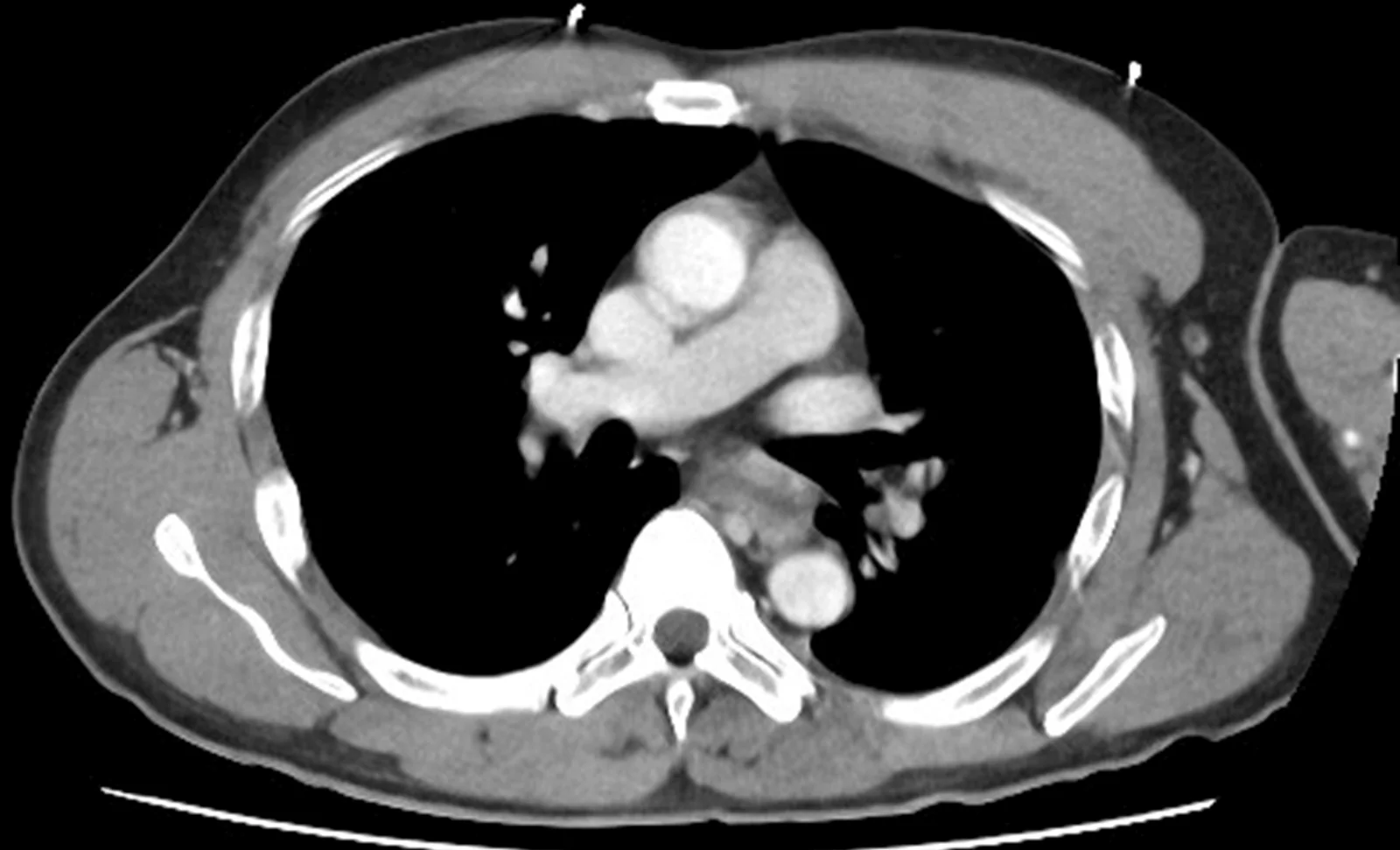
Contrast-enhanced axial CT image of the chest Representative contrast-enhanced axial CT image of the chest obtained during evaluation for occult causes of severe lactic acidosis. No acute thoracic abnormality or infective focus was identified on CT imaging.

**Figure 2:**
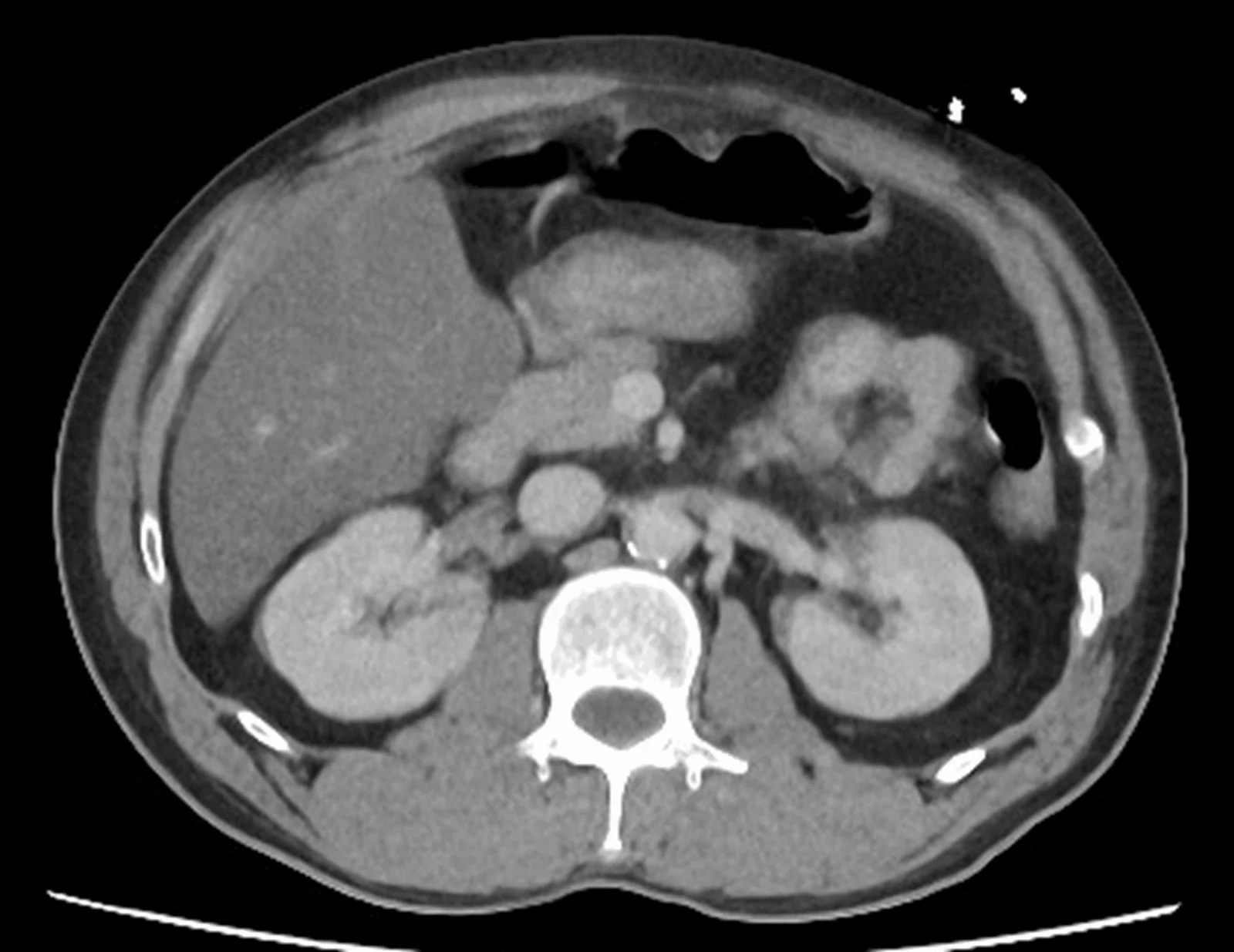
Contrast-enhanced axial CT image of the abdomen Representative contrast-enhanced axial CT image of the abdomen obtained during evaluation for intra-abdominal causes of severe lactic acidosis. No acute intra-abdominal abnormality, bowel ischaemia, or infective focus was identified on CT imaging.

Contrast-enhanced computed tomography of the chest, abdomen, and pelvis did not demonstrate an infective focus, bowel ischaemia, intra-abdominal pathology, or other acute abnormality to explain the severity of metabolic acidosis. Representative axial CT images are shown in Figure 1 and Figure 2.

The patient initially received 1.5 L of intravenous 0.9% sodium chloride during the first 90 minutes, followed by maintenance intravenous fluids that included Hartmann’s solution and 0.9% sodium chloride. Intravenous thiamine supplementation was administered early because of the history of heavy alcohol use and risk of nutritional deficiency. Following treatment, his tachycardia improved from 114 beats/min to 88 beats/min, and his tachypnoea improved over the next few hours. Serial venous blood gas analyses demonstrated rapid improvement in acidaemia and lactate clearance, with lactate decreasing from greater than 20 mmol/L on admission to 4.6 mmol/L by 10 hours and 1.6 mmol/L by 16 hours. The pH normalised to 7.37 by 12 hours. Blood ketones also improved over the first 24 hours. The serial blood gas trend is shown in Table 2.

**Table 2:** Serial biochemical and acid–base parameters during the first 24 hours after admission pH is unitless. Lactate, bicarbonate, anion gap, and blood ketones are reported in mmol/L. “—” indicates not measured or not available.

Time from admission	pH	Lactate, mmol/L	Bicarbonate, mmol/L	Anion gap, mmol/L	Blood ketones, mmol/L
1 hour	6.86	>20	5.9	40	—
2 hours	6.96	19.5	2.7	39	6.5
4 hours	6.92	18	3.5	37	—
6 hours	7.03	11.2	2.6	29	4.7
8 hours	7.10	9.2	4.3	29	4.5
10 hours	7.29	4.6	9.3	24	4
12 hours	7.37	3.3	14.2	—	3.7
16 hours	7.42	1.6	19.9	—	1.7
18 hours	7.43	1.6	20.4	—	0.6
20 hours	7.47	1.4	20.4	—	1.4
24 hours	7.49	1.5	22.1	—	—
Reference range	7.35 - 7.45	0.5 - 2.0	22 - 29	12 - 20	0 - 0.6

Given the history of heavy alcohol exposure, elevated serum ethanol concentration, profound lactic acidosis, marked ketonaemia, and the absence of another immediately apparent cause following initial evaluation, a clinical diagnosis of alcohol-associated lactic acidosis with concomitant ketoacidosis was made. The patient made a full clinical and biochemical recovery and was discharged home on day four. He was monitored for alcohol withdrawal during admission, and onward alcohol-related care was arranged in accordance with local clinical pathways.

## Discussion

This case describes a rare and severe presentation of alcohol-associated lactic acidosis (AALA) with concomitant ketoacidosis, characterised by profound acidaemia and a lactate level greater than 20 mmol/L. Although AALA is uncommon, it is clinically important because it can mimic other life-threatening causes of high anion gap metabolic acidosis. The diagnosis should therefore be considered only after careful evaluation for alternative or contributing aetiologies, including sepsis, shock, seizures, mesenteric ischaemia, diabetic or alcoholic ketoacidosis, hepatic failure, renal failure, salicylate toxicity, metformin-associated lactic acidosis, and toxic alcohol ingestion [1,2,5,6].

Alcoholic ketoacidosis was an important differential diagnosis and co-existing process in this case. Alcoholic ketoacidosis typically occurs after heavy alcohol intake followed by reduced oral intake, vomiting, and starvation, and is characterised by high anion gap metabolic acidosis, elevated ketones, and normal or low blood glucose [5]. This patient had several features consistent with alcoholic or starvation ketoacidosis, including vomiting, normal blood glucose, and marked ketonaemia. However, isolated alcoholic ketoacidosis would not fully explain the lactate concentration greater than 20 mmol/L. Marked lactate elevation in the setting of ethanol intoxication should prompt evaluation for alternative or additional causes [3]. In this case, the very high lactate level, elevated ethanol concentration, absence of an alternative cause following initial evaluation and rapid lactate clearance with supportive treatment favoured alcohol-associated lactic acidosis as the dominant process, with concomitant ketoacidosis contributing to the overall high anion gap metabolic acidosis.

In this case, the severity of acidaemia and hyperlactataemia prompted urgent assessment for these alternative diagnoses. Sepsis was considered less likely despite mild leucocytosis, as the patient was afebrile, had a normal C-reactive protein level, and no infective focus on contrast-enhanced computed tomography of the chest, abdomen, and pelvis. Sustained systemic hypoperfusion was not supported by the absence of hypotension, normal capillary refill time, preserved peripheral pulses, and satisfactory urine output. Serum creatinine was only mildly elevated, with preserved estimated glomerular filtration rate, making renal failure unlikely to explain the severity of acidosis, with serum creatinine levels normalising by 12 hours. Although liver biochemistry showed transaminitis with aspartate transaminase predominance, preserved bilirubin, albumin, and coagulation profile argued against acute hepatic failure. Blood glucose was normal, while ketones were markedly elevated, supporting concomitant alcoholic or starvation ketoacidosis rather than diabetic ketoacidosis [5,7]. Negative paracetamol and salicylate levels, methanol within the laboratory reference range, and normal cross-sectional imaging further reduced the likelihood of common toxicological or ischaemic causes.

The pathophysiology of AALA is multifactorial. Ethanol metabolism increases the reduced nicotinamide adenine dinucleotide to nicotinamide adenine dinucleotide ratio (NADH/NAD+), favouring conversion of pyruvate to lactate and impairing gluconeogenesis [4,5]. Heavy alcohol use may also contribute through poor nutritional intake, starvation ketosis, and thiamine deficiency. Thiamine is an essential cofactor for pyruvate dehydrogenase, and deficiency may divert pyruvate metabolism towards lactate production [8]. In this patient, both ethanol-related lactate accumulation and marked ketosis likely contributed to the severe high anion gap metabolic acidosis. However, the extreme lactate elevation greater than 20 mmol/L and rapid lactate clearance suggest that lactic acidosis was a major component of the presentation.

Marked lactate elevation is unusual in uncomplicated acute ethanol intoxication. MacDonald et al. reported that when lactic acidosis is identified in patients with ethanol intoxication, clinicians should actively evaluate for alternative or contributing causes [3]. However, critically ill patients with AALA may present with markedly elevated lactate levels and can have favourable outcomes when lactate clearance is rapid [2]. Yang et al. reported that peak lactate level was not independently associated with survival in AALA, whereas survivors demonstrated better lactate clearance [2]. Similar favourable outcomes have been reported in cases of profound alcohol-related or ethanol-induced lactic acidosis managed with supportive treatment and metabolic correction [9,10]. Because this is a single case report, the precise relative contributions of alcohol-associated lactic acidosis, concomitant ketoacidosis, dehydration and possible thiamine deficiency cannot be determined; however, the rapid biochemical recovery is consistent with previous case reports of reversible alcohol-related lactic acidosis [7,9,10].

Management is primarily supportive and should focus on haemodynamic stabilisation, correction of metabolic abnormalities, treatment of nutritional deficiency, and close monitoring. Intravenous fluid resuscitation may improve lactate clearance, particularly when dehydration or reduced effective circulating volume contributes. In patients with severe metabolic acidosis, ongoing fluid choice should be individualised, as excessive 0.9% sodium chloride administration may contribute to hyperchloraemic metabolic acidosis and complicate interpretation of acid-base recovery. Thiamine should be administered early in patients with heavy alcohol use or suspected malnutrition because of its role in pyruvate metabolism [8]. In this case, intravenous fluids, early intravenous thiamine, and intensive care monitoring were associated with rapid biochemical recovery. Lactate decreased from greater than 20 mmol/L on admission to 4.6 mmol/L by 10 hours and normalised by 16 hours, while the pH normalised within 12 hours. The patient recovered without vasopressor support, ventilatory support, or renal replacement therapy.

This case highlights that severe high anion gap metabolic acidosis in patients with significant alcohol exposure may be multifactorial, with lactic acidosis, ketosis, dehydration, and nutritional deficiency contributing to varying degrees. AALA should be considered when profound hyperlactataemia occurs in this context, particularly after life-threatening causes have been appropriately evaluated. Prompt supportive treatment, thiamine replacement, and close biochemical monitoring may lead to rapid clinical and metabolic recovery.

## Conclusions

Alcohol-associated lactic acidosis is an uncommon but clinically important cause of severe high anion gap metabolic acidosis in patients with significant alcohol exposure. This case highlights that profound hyperlactataemia may occur in the setting of heavy alcohol intake and may co-exist with ketoacidosis, even in the absence of shock, hypoxia, sepsis, or major organ failure.

Profound lactic acidosis in patients with alcohol exposure should prompt urgent and systematic evaluation for alternative or contributing aetiologies, including sepsis, hypoperfusion, seizures, ischaemia, renal or hepatic failure, diabetic ketoacidosis, and toxic ingestion, before alcohol is considered the primary cause. Early recognition, supportive treatment with intravenous fluids and thiamine supplementation, and close biochemical monitoring may be associated with rapid clinical and metabolic recovery.
